# Prognostic Value of Lordosis Decrease in Radiographic Adjacent Segment Pathology After Anterior Cervical Corpectomy and Fusion

**DOI:** 10.1038/s41598-017-14300-4

**Published:** 2017-10-31

**Authors:** Yin Liu, Na Li, Wei Wei, Jing Deng, Yuequn Hu, Bin Ye, Wei Wang

**Affiliations:** 1 Department of Radiology, the Third Xiangya Hospital, Central South University, Changsha, P.R. China; 2 Laboratoire de Biomécanique Appliquée, MRT24 IFSTTAR-Aix-Marseille Université, Bd. P. Dramard, Faculté de Medecine secteur-Nord, Marseille, 13916 France; 3 Xiangya School of Public Health, Central South University, Changsha, P.R. China

## Abstract

While cervical lordosis alteration is not uncommon after anterior cervical arthrodesis, its influence on radiological adjacent segment pathology (RASP) is still unclear. Biomechanical changes induced by arthrodesis may contribute to ASP onset. To investigate the correlation between cervical lordosis decrease and RASP onset after anterior cervical corpectomy and fusion (ACCF) and to determine its biomechanical effect on adjacent segments after surgery, 80 CSM patients treated with ACCF were retrospectively studied, and a baseline finite element model of the cervical spine as well as post-operation models with normal and decreased lordosis were established and validated. We found that post-operative lordosis decrease was prognostic in predicting RASP onset, with the hazard ratio of 0.45. In the FE models, ROM at the adjacent segment increased after surgery, and the increase was greater in the model with decreased lordosis. Thus, post-operative cervical lordosis change significantly correlated with RASP occurrence, and it may be of prognostic value. The biomechanical changes induced by lordosis change at the adjacent segments after corpectomy may be one of the mechanisms for this phenomenon. Restoring a well lordotic cervical spine after corpectomy may reduce RASP occurrence and be beneficial to long-term surgical outcomes.

## Introduction

Early surgery may effectively change the unfavorable prognosis of patients with cervical spondylotic myelopathy (CSM)^1–4^. Anterior cervical corpectomy and fusion (ACCF) is a commonly performed surgery in treating CSM, especially multilevel CSM, which may allow a better decompression of the neural element and less graft-bone surfaces for fusion comparing to 2-level discectomy^5,6^. Donor comorbidity by harvesting autologous bone graft, such as local pain or fracture, can also be avoided as well^7^.

As the prevalence of CSM increases and the age of patients treated by surgery decreases, special attention is being paid to the long-term surgical outcome. Adjacent segment pathology (ASP) is an important issue as it occurs in one out of every 3–4 patients within 10 years after anterior arthrodesis, and is also one of the most common reasons for revision surgery^8^. However, ASP in CSM patients treated by corpectomy is less studied, and its pathogenetic mechanism remains uncertain. In addition to the effects of aging, heredity and many other factors, the role of biomechanical factors in disc degeneration is widely recognized. Abnormal loading such as vibration, torsion and compression may affect synthetic activity and extracellular matrix molecular expression in the disc^9^. Biomechanical loading shifts, such as an increase of segmental range of motion (ROM), intradiscal pressure, and stiffness to adjacent segments after arthrodesis were also consistently observed in many experimental and clinical studies^10–13^, and may play an important role in ASP onset.

Cervical lordosis is a unique morphological characteristic of the cervical spine and may help to optimize the biomechanical loading in this region. As one of the factors to consider in surgical planning^4^, surgeons may choose an anterior over a posterior approach in cases of alignment deformity to achieve a better decompression outcome. Post-surgical malalignment might be associated with ASP occurrence^14–18^, though irrelevance was also noted^19,20^. As the range of normal cervical lordosis is large and the ratio of kyphosis is low, the influence of lordosis change may be underestimated if not directly compared to the patient’s pre-surgical condition. This underestimation may contribute to the controversy in previous studies. In addition, as proposed by Benzel *et al*.^21^, once the cervical lordosis decreases, moments imposed on the whole cervical spine, the instaneous axis of rotation (IAR) and lever arm length (starting from the IAR), may be altered, and the biomechanical shifting at adjacent segments may be subsequently affected.

To elucidate the role of cervical lordosis change after ACCF in ASP onset, we examined the relationship between cervical lordosis change and RASP onset in a group of CSM patients treated by ACCF. We hypothesized that post-surgical cervical lordosis decrease might affect the biomechanical loading shift at the adjacent segment, which in turn might contribute to ASP onset. Therefore, we developed a finite element model to simulate post-surgical cervical spine with normal and decreased lordosis to test our hypothesis. The surgical maneuver and lordosis (C2-7 Cobb angle) change simulated in the post-surgery finite element models were determined according to the patients we studied.

## Result

### Clinical Outcomes

In the 80 patients included, RASP was present in 37 patients. The demographics and clinical information for both the RASP and non-RASP groups are listed in Table 1. No statistical difference was found between the two groups, excepting a longer follow-up time in the RASP group (36.8 ± 20.7 vs 23.7 ± 12.9 months). Pre-operative, post-operative, and follow-up JOA scores are also listed in Table 1. Post-operative and follow-up JOA scores were significantly improved in both groups. The follow-up JOA score was significantly lower than the post-operative JOA score in the RASP group, but higher in the non-RASP group.

**Table 1:** Demographic and Clinical Information of Patients in RASP and Non-RASP groups.

Age	RASP group (n = 37)	Non-RASP group (n = 43)	P value
	50.3 ± 9.3	51.0 ± 8.7	0.87
Gender(male)	18	19	0.69
Corpectomy			
C4	2	5	0.55
C5	17	18	0.71
C6	13	12	0.48
C4-5	2	3	0.86
C5-6	3	5	0.88
Follow-up period(month)	36.8 ± 20.7	23.7 ± 12.9	<0.01
Pre-operative JOA score	10.8 ± 3.1	10.7 ± 2.9	0.82
Post-operative JOA score	14.3 ± 2.4*	14.4 ± 2.5*	0.91
Follow-up JOA score	13.9 ± 2.3**	14.6 ± 2.7**	0.23
Post-operative RR(%)	60.3 ± 29.8	61.6 ± 25.3	0.83

^*^P < 0.05 comparing to pre-operative JOA score.

**P < 0.05 comparing to post-operative JOA score.

**Figure 1:**
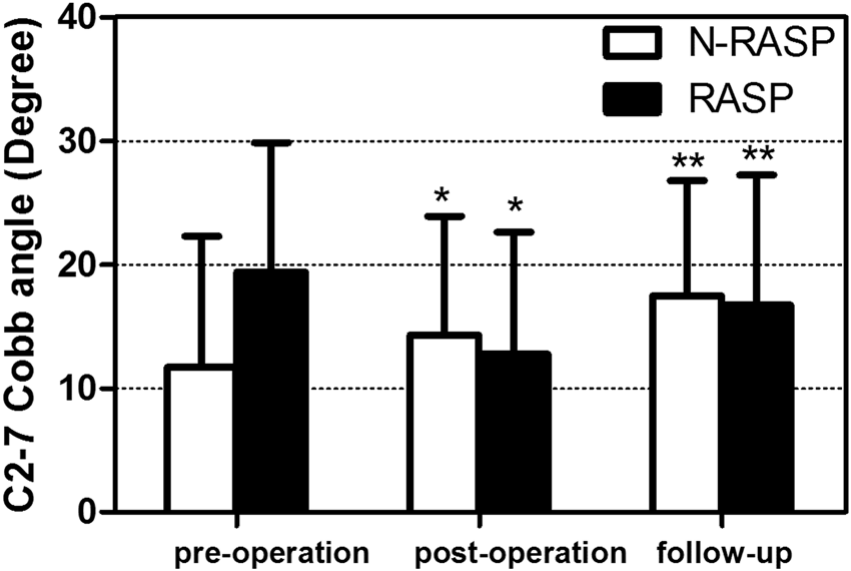
Pre-operative, post-operative, and final follow up cervical Cobb angle change. *p < 0.05 comparing to pre-operation using paired *t* test. **P < 0.05 comparing to pre-operation and post-operation using paired *t* test.

Table 2 shows the global alignment of both groups before and after operation. Figure 1 shows the average C2-7 Cobb angle in both groups before and after surgery. The post-operative C2-7 and fused segment Cobb angle change were both significantly correlated with RASP onset. After adjustment of confounding factors of age, gender, operation location, fused segment, pre-operative alignment, and operating surgeons, a Cox regression analysis was further performed to evaluate the prognostic value of post-surgical lordotic angle change and the results showed that only the post- surgery C2-7 Cobb angle change was significantly associated with RASP onset, with the hazard ratio of 0.454 (Table 3).

**Table 2:** Cervical alignment change before and after operation.

After surgery			Total
Before surgery	Lordosis	Hypolordosis	
Lordosis	45	16	61
Hypolordosis	9	7	16
Kyphosis	1	2	3
Total	55	25	80

**Table 3:** Cox regression analysis for the predictive value of post-operation lordosis angle change in RASP occurrence.

Parameter	*β*	Wald *χ* ^2^	P value	Hazard Ratio
C27CA-c	−0.518	4.262	0.039*	0.454 (0.297–0.692)
FCA-c	0.111	2.139	0.144	1.118 (0.963–1.298)

C27CA-c: Post-surgery C2-7 Cobb angle change; FCA-c: Post-surgery fused segment Cobb angle change.

### Post-surgical FE model validation

**Figure 2:**
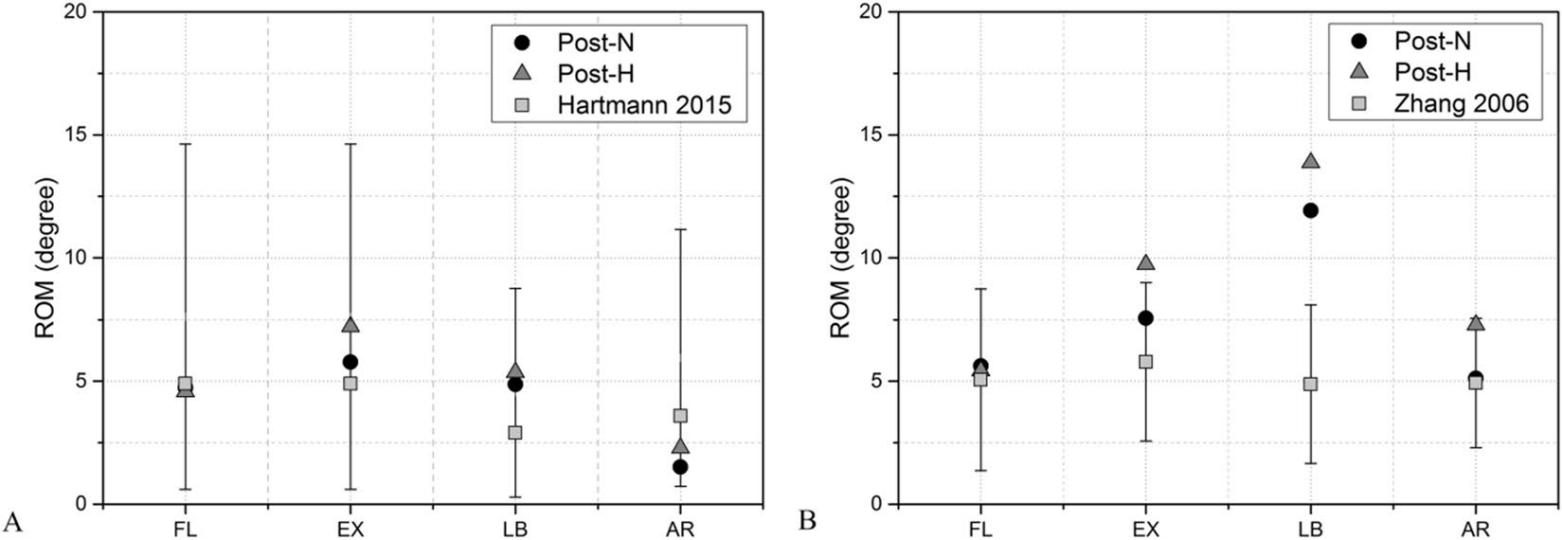
Validation of post-operation models with normal and decreased lordosis after C5 subtotal corpectomy and fusion.

ROM of the operated segments (C4-6) in both post-surgical models were validated and matched well with Hartmann (2015) in all motions. It also matched well with results obtained from Zhang (2006) in flexion, extension, and axial rotation, but C4-6 ROM in post-H was 7.6% larger in extension, In addition, it was 48% and 70% larger in post-N and post-H during lateral bending (Fig. 2).

### Adjacent segment response in post-surgical models with different lordotic angles

**Figure 3:**
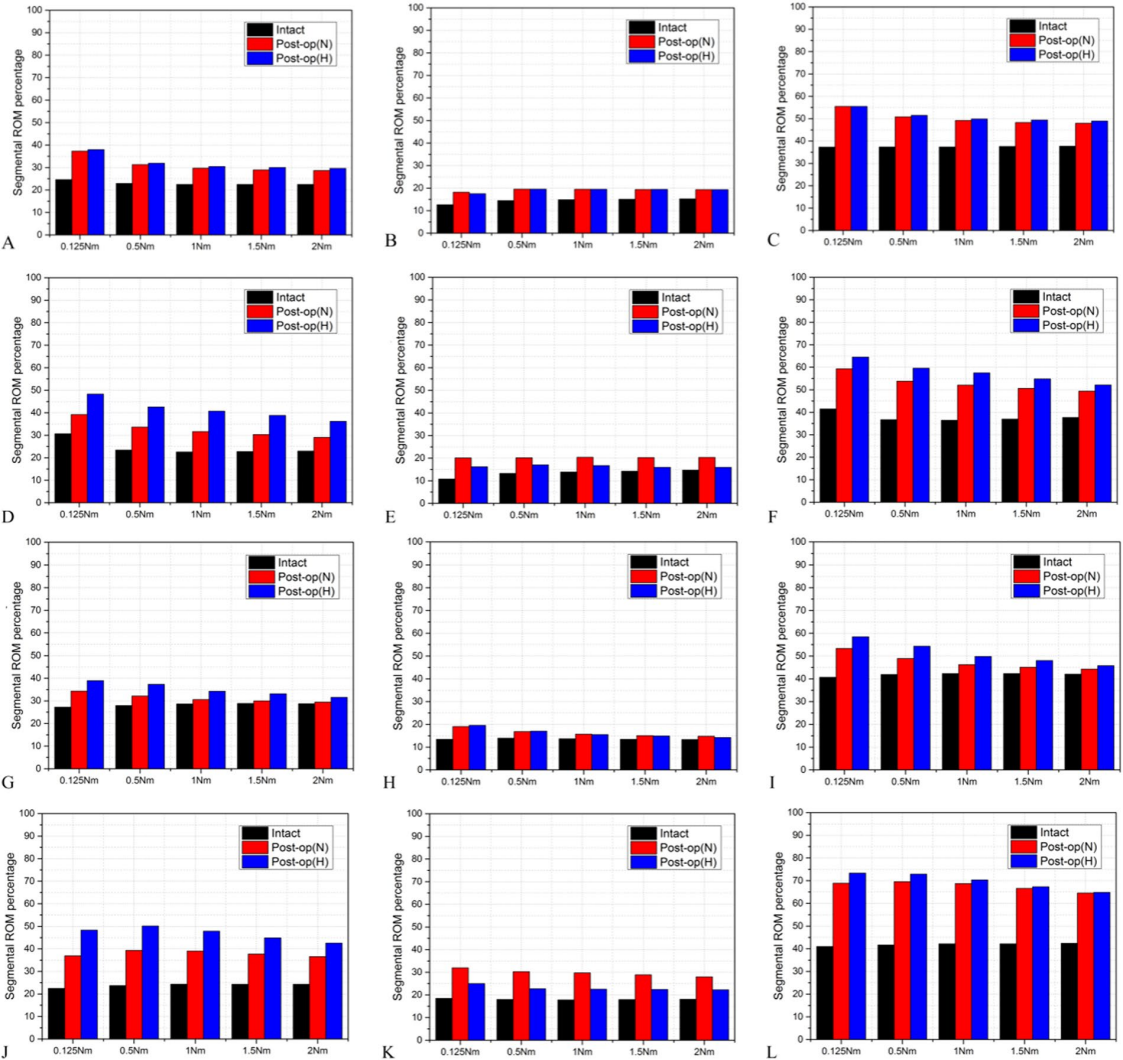
The segmental ROM percentage at superior (left row), inferior (middle row) and both (right row) adjacent segment in the intact model, post-operation models with normal lordosis (post-N) and decreased lordosis (post-H) in flexion (**A**–**C**), extension (**D**–**F**), lateral bending (**G**–**I**) and axial rotation (**J**–**L**) under moment-control loading.

Under moment-control loading, the percentage of adjacent segment ROM was increased consistently in post-surgery models; in particular, the superior adjacent segment (SAS) was more obvious than the inferior adjacent segment (IAS), as shown in Fig. 3. The ROM percentage increase was consistently greater in post-H model in extension, lateral bending and axial rotation, especially at the SAS. However, the ROM percentage change was not obvious in flexion in both post-surgery models.

**Figure 4:**
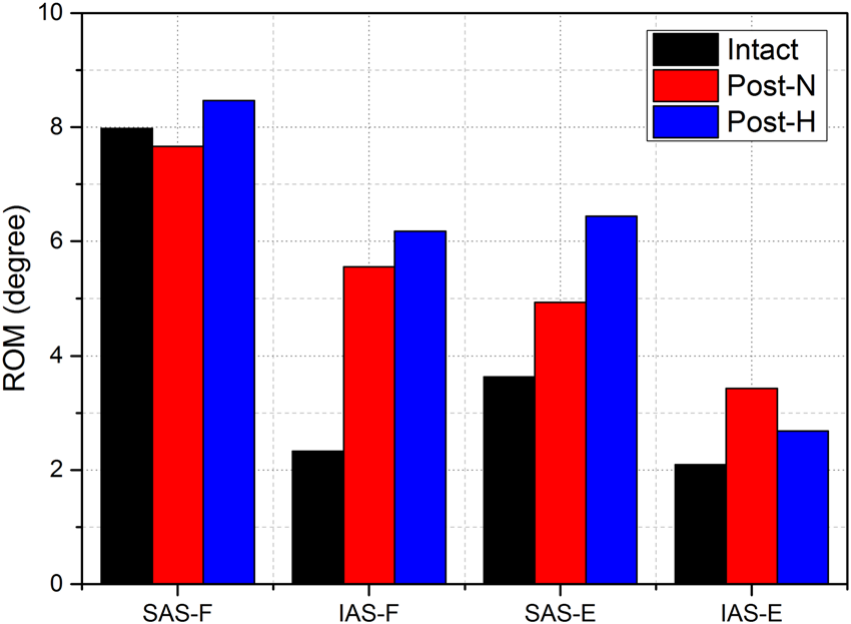
The superior and inferior segment ROM in intact and post-operation models with different lordosis in flexion and extension under displacement-control loading. (SAS: superior adjacent segment; IAS: inferior adjacent segment; F: flexion; E: extension.)

**Figure 5:**
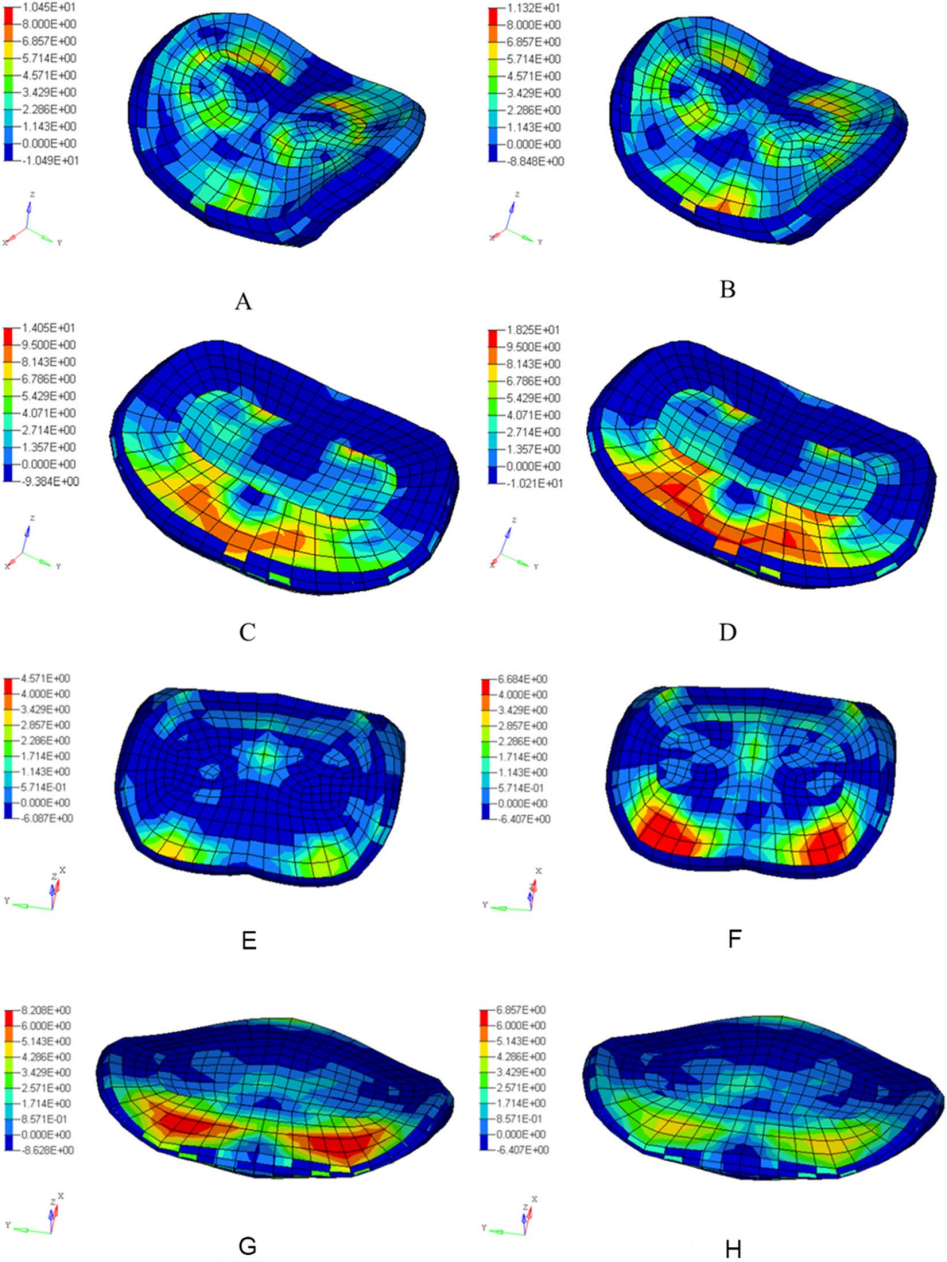
Comparison of intradiscal pressure distribution of superior (**A, B** and **E, F**)and inferior (**C, D**, and **G, H**) adjacent segment in flexion (**A–D**) and extension  (**E–H**) under displacement-control loading. (**A**,**C,E,G**: post-N model; **B**,**D,F,H** post-H model).

Under displacement-control loading, adjacent segmental ROM increase was greater in post-H model during flexion, especially the IAS (Fig. 4). The increase percentage at the SAS, the IAS, and both was −4%, 138.36% and 28.3% in post-N, and 6.1%, 165.08% and 42.07% in post-H. The intradiscal pressure was obviously larger in the adjacent segments of post-H, and the maximum pressure at the SAS of post-N and post-H was 10.45 MPa and 11.32 MPa, while the IAS was 14.05 MPa and 18.25 MPa, respectively(Fig. 5 A–D). During extension, the SAS ROM increase was greater, and the IAS ROM increase was smaller in post-H (Fig. 4). The increase percentage at the SAS, the IAS, and both AS was 35.78%, 63.78%, 46.02% in post-N, and 77.22%, 28.23%, 59.30% in post-H. Similarly, the intradiscal pressure was greater at the SAS but smaller at the IAS in post-H, with the maximum pressure at the SAS of post-N and post-H at 3.63 MPa and 6.68 MPa. The maximum pressure at the IAS was 8.21 MPa and 5.16 MPa, respectively  (Fig. 5 E–H).

## Discussion

According to the clinical manifestation ASP can be classified into RASP and CASP. Primarily diagnosed with imaging examination, RASP is relatively objective and may be graded. However, though CASP tend to be more often in patients with RASP^7,12,14^, the correlation between RASP and CASP remains elusive. Similar with previous studies, the prevalence of RASP was 46.25% in the patients we studied, and the follow-up JOA scores were significantly lower in RASP group, though the immediate post-surgery RR was not significantly different between the 2 groups. The correlation between RASP and CASP was not confirmed in this study, which may be influenced by the relatively short follow-up time and scarcity of CASP.

Previous clinical studies indicated that post-operative cervical lordosis may be relevant to the therapeutic outcome of cervical arthrodesis^14,22,23^, but its influence to ASP occurrence was still uncertain. Kastuura *et al*.^14^ and Faldini *et al*.^17^ found that ASP in lordotic patients was significantly lower than non-lordotic patients after arthrodesis. In both studies, the C2-7 Cobb angle significantly correlated with ASP occurrence. However, in the studies of Kulkarni *et al*.^16^ and Park *et al*.^18^, the post-surgical C2-7 Cobb angle was not significantly correlated with ASP, though both studies found that ASP was significantly higher in non-lordotic patients after surgery. Others have also shown that there was no correlation between them^19,20^. Most of these studies, however, only examined the patients’ post-surgical lordosis conditions and largely ignored their pre-operative status. In this study, we found that post-operative C2-7 Cobb angles significantly decreased in the RASP group, but significantly increased in the non-RASP group, both in the immediate post-operative period and the final follow-up. As suggested by Cox regression analysis in this study, C2-7 Cobb angle change was significantly associated with RASP onset, even with a mild degree. Thus, maintaining a well lordotic cervical alignment after ACCF may be beneficial in reducing post-surgical RASP onset.

The mechanism for such influence is unclear. The role of biomechanical factors in disc degeneration has been recognized, as consistent and repetitive loading mimicking rigorous daily activities may induce disc damages^9^. Similarly, segmental ROM and intradiscal pressure were increased at adjacent segments after ACF, demonstrated by both *in vitro* ^10,11^ and *in vivo* studies^12,13^. Thus, post-surgical biomechanical loading shift was considered an important factor in ASP pathogenesis. According to Benzel *et al*.^21^, it was speculated that once the lordosis changes, the instaneous axis of rotation (IAR) location, lever arm length and subsequent moment imposed on the cervical spinal structures may be altered. The loading was increased at the anterior column of the cervical spine, while the posterior annulus and Sharpey’s fibers might be extended to detach from the endplate, with an increase of uncovertebral and facet joints loading. However, it is difficult to precisely obtain the biomechanical parameter change in either animal or cadaver study by accurately change the cervical alignment. In this study, we innovatively calculated the qualitative change in adjacent segment after ACCF, using finite element models in which only the lordotic angle differed. The results showed that intradiscal stress and ROM at adjacent segment demonstrated greater change in less lordotic post-surgical model, which supported Benzel’s hypothesis and may provide a clue for the mechanism of ASP occurrence after ACCF. The lordosis decrease, even if a mild degree, may cause biomechanical loading alteration at adjacent segment which may subsequently contribute to RASP onset.

Numerical modeling has been used in studies of cervical spine surgery^24^ and cervical lordosis abnormality^25^ as an invaluable tool and complement to the experimental study of biomechanics. Extensive validation is necessary for FE modeling studies to add confidence in the accuracy of results. For this reason, in addition to intact model validation, we also validated the post-surgical models with previous cadaveric studies. Except for a higher ROM in lateral bending than found in the results of Zhang *et al*.^26^, both validations for the intact model and post-surgical models were in agreement with previous studies. As the intra- and inter-deviations in *in vitro* studies were large, and the result reported by Zhang *et al*. was lower than other *in vitro* studies^27,28^, we believe that the models developed in this study were biofidelic, and may provide new clues for future studies.

It is still controversial as to whether moment-control or displacement-control is closer to physiological conditions, and so we adopted both loading schemes in this study. Both post-operative models showed an increase of segmental ROM and intradiscal pressure at adjacent segments, but the increase was more obvious in the post-H model under both moment and displacement-control loading conditions. These results were in agreement with previous *in vitro* studies of Hwang *et al*.^29^ and Wang *et al*.^30^. They both found that cervical lordosis change might have induced the biomechanical loading or motion difference. In the study of Hwang *et al*.^29^, the segmental lordosis was changed by spacers with different sizes, and the ROM of the adjacent segment was significantly increased in the slight lordosis increase group (small spacer); but it was not significantly different in the group with larger spacers. However, the difference was not significant once the pre-surgical lordosis was adjusted. It may be because the fused segment Cobb angle was not significantly relevant, as shown in this study, but this still awaits validation in future studies as sampling bias could not be excluded.

There are some limitations to this study. Firstly, the relatively small sample size and the nature of retrospective study may add bias to the clinical information collected and thus limit the generalizablity of this study. Further study was still warranted. Secondly, although kyphotic change was generally considered abnormal and possibly necessitating intervention, the incidence in this cohort and previous studies was low. Therefore, we only studied the influence in case of cervical lordosis decrease (straightening) in FE analysis while other alignment deformities were not included. In addition, other measurements of cervical alignment such as sagittal vertical axis (SVA) and chin-brow vertical angle (CBVA), which may be important to surgical outcome^31^, were not examined in this study as they were difficult to obtain retrospectively and might reflect long-term adaptation of the whole spine. Furthermore, the FE models of this study only predicted the biomechanical responses of osseous and articulation structures, while active muscle response and neuro-muscular coupling of humans could not be accurately presented in current FE modeling and cadaver experiments. Similarly, as the surgery we simulated was C5 subtotal corpectomy and fusion, the results should not be extrapolated to reflect the biomechanical change at the adjacent segment in other surgical approaches, as it may vary with surgery location and fused segment number.

In summary, our study demonstrated that post-operative cervical lordosis decrease significantly correlates with RASP occurrence in CSM patients treated by ACCF and may be of prognostic value. The difference in ROM and IDP increase at adjacent segments after ACCF observed in post-surgery models with normal and decreased alignment may be one of the underlying mechanisms for the phenomenon. Therefore, restoring a well lordotic cervical spine after ACF may improve long-term surgical outcome and reduce the incidence of RASP.

## Materials and Method

### Patients

144 CSM patients treated by ACCF and received imaging examinations in our department between January 2008 and December 2013 were retrospectively studied. Inclusion criteria were: a diagnosis of CSM which was treated by ACCF, a minimum follow-up of 1 year, and the availability of complete clinical and imaging information. To reduce other confounding factors, patients were excluded if there were prior cervical surgeries or if a discectomy or if a posterior approach in cervical surgery; and were concurrent non-degenerative conditions, such as cervical spine fracture, active inflammation or infection, connective tissue disease, neoplasm, and congenital abnormalities. Finally, 80 CSM patients treated by ACCF were included. All of the patients had titanium mesh cage with autologous bone graft and anterior cervical plating fixation.

Of those patients, 62 patients were excluded due to loss of follow-up or incomplete imaging data, and 2 patients died within 2 years after surgery due to unrelated conditions. Therefore, a total of 80 patients (37 male, 43 female) were included for analysis in this retrospective study, and all of them received the surgery in the Department of Orthopeadics and Spine Surgery, the Third Xiangya Hospital, Central South University. The surgery was performed by the same group of surgeons. This study was approved by the ethics committee of the Third Xiangya Hospital, Central South University, and all methods were in accordance with the relevant guidelines and regulations. Written and/or oral informed consent was obtained from each of the patients.

The average patient age at surgery was 50.5 ± 8.9 years (range: 29–75 years old), and the average follow-up period was 29.8 ± 18.1 months (range: 12–88 months). 67 patients had a 1-level and 13 patients had a 2-level anterior cervical corpectomy and fusion.

### Imaging evaluation

All patients had a radiological examination of the cervical spine pre-operatively, post-operatively (2–4 weeks after surgery), and at final follow-up (at least 1 year after surgery). Imaging included frontal and neutral lateral radiographs of the cervical spine. Pre-operative and follow-up MRI and/or CT images of the cervical spine were also obtained. RASP was diagnosed when new or progressed degeneration was found at an adjacent segment, including disc space narrowing, osteophyte enlargement, endplate sclerosis, uncoarthrosis and facet joint degeneration by radiography and/or disc protrusion on CT/MRI. The C2-7 Cobb angle and fused segment Cobb angle were measured on pre-operative, post-operative, and follow-up lateral radiographs of the cervical spine. The global sagittal alignment of the cervical spine was further classified as lordotic, straightened, kyphotic, or sigmoid, according to the Toyama classification^32^. All the images were evaluated by two experienced radiologist (Ye B. and Liu Y.) who were blind to patients’ clinical outcome, twice within a month, and the average were used for analysis. RASP diagnosis for each patient was agreed by both radiologists.

### Clinical evaluation

Demographic data, clinical diagnosis and surgery information were obtained from electronic medical records, including the patient’s age at operation, gender, surgery information, and follow-up period. Surgical outcome was evaluated using recovery rate (RR), which was calculated from Japanese Orthopedica Association (JOA) scores before and after surgery (within 1 month after surgery and at final follow-up) using the formula proposed by Hirabayashi *et al*.^33^. Operative results and postoperative progression of ossification were measured for patients with ossification of the cervical posterior longitudinal ligament. [RR(%) = (postoperative JOA score-preoperative JOA score)/(17-preoperative JOA score) × 100].

### Finite element model development and loading condition

**Figure 6:**
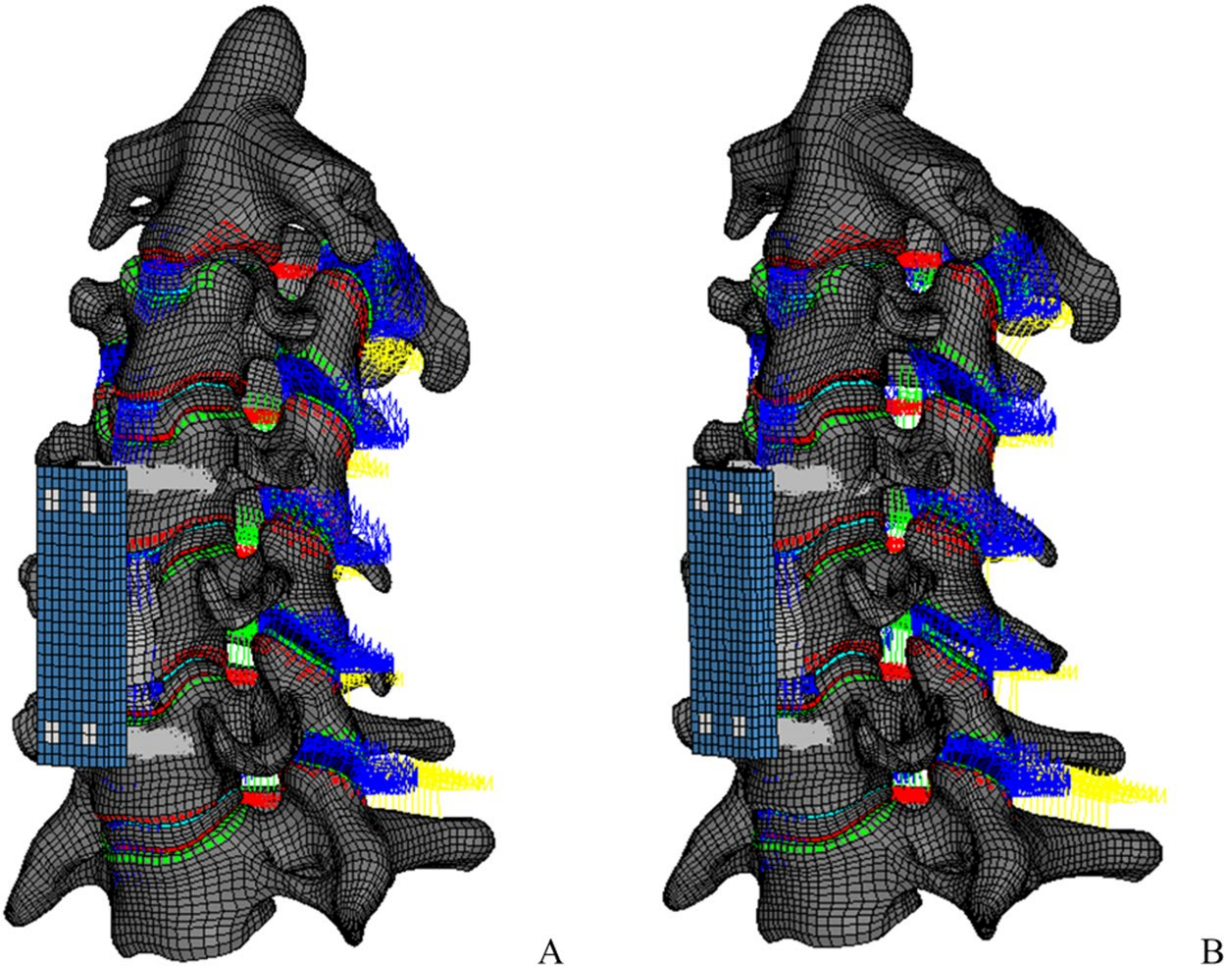
The finite element models of C2-7 cervical spine after C5 subtotal corpectomy reconstruction with bone graft and anterior plate fixation. The post-operation models showed normal lordosis (Post-N model) (**A**) and decreased lordosis (Post-H model) (**B**).

An anatomical detailed finite element model of human C0-7 cervical spine was developed previously^34^. As most *in vitro* and numerical studies on ASP used the subaxial cervical spine and there was no upper cervical fusion in the patients studied above, we only used a C2-7 model (intact model) in the surgery simulation. The post-operative models with normal lordosis (post-N) and decreased lordosis (post-H) were developed on the basis of the intact model (Fig. 6). The surgical intervention simulated in this study was a C5 subtotal corpectomy with the reconstruction of a graft implant and anterior cervical plating, which was also the most common surgery performed in the clinical sample. To be as close to a clinical scenario as possible, the postsurgical models were developed by removing the central part of the C5 vertebral body, the nucleus and surrounding annulus fibrosis of the C4-5 and C5-6 disc, as well as the ALL and PLL of the C4-5 and C5-6. The lateral peripheral annulus of discs, bilateral facet joints, uncovertebral joints and posterior spinal element remained intact. The osseous graft was placed anteriorly to the center of the resected C5, in-between the undersurface of C4 and the upper surface of C6, with rigid fusion of the endplates. A titanium plate (40 × 13 × 2 mm) was placed in front of the C4-6 vertebral body with a rigid screw in C4 and C6. According to neutral lateral cervical radiography of the typical patients in the RASP group, a post-surgical model with decreased lordosis was developed by slightly changing the vertebral positions in the post-N model  (Fig. 6). The C2-7 Cobb angle for post-N and post-H models were 22° and 13° respectively, which was close to the average value for pre-operative and post-operative Cobb angle in RASP group. The material properties of the models and instrumentation are listed in Table 4.

**Table 4:** Material Properties of Cervical Spine Finite Element Modeling.

Tissue names	Element type	Material Type	Material Parameters	Reference
Cortical bone	Shell	power-law plasticity	ρ = 1.61e-9 t/mm^3^, E = 16700 MPa, μ = 0.3, K = 354.8 MPa, N = 0.2772	37
Cancellous bone	hexahedron	power-law plasticity	ρ = 8.77e-10 t/mm^3^, E = 291 MPa, μ = 0.3, K = 5.7 MPa, N = 0.2741	38
Endplate	Shell	power-law plasticity	ρ = 1.61e-9 t/mm^3^, E = 5600 MPa, μ = 0.3, K = 153.2 MPa, N = 0.2772	39
Matrix of annulus fibrosus	hexahedron	Hill Foam	m = 3, n = 2, C1 = 2.1857 MPa, b1 = 1, C2 = −2.36 MPa, b2 = 2, C3 = 0.891 MPa, b3 = 3	40
Annulus fibrosus fibers	Shell	Fabric	Strain-Stress Curve	41
Nucleus	hexahedron	General viscoelastic	N = 4, K = 1.72 Gpa G_1_ = 0.5930 kPa, β_1_ = 0.001477 1/s, G_2_ = 0.6763 kPa, β_2_ = 0.061524 1/s, G_3_ = 0.9516 kPa, β_3_ = 1.017893 1/s, G_4_ = 2.0384 kPa, β_4_ = 13.20041 1/s,	42,43
Cartilage endplate	hexahedron	Isotropic elastic	ρ = 1.36e-9 t/mm^3^, E = 25 MPa, μ = 0.4	44
Facet articular cartilages	Hexahedron	Isotropic elastic	ρ = 1.36e-9 t/mm^3^, E = 10.4 MPa, μ = 0.4	45
Ligaments	Beam	Non-linear	Displacement-Force Curve	46,47
Bone graft	hexahedron	Power-law plasticity	ρ = 8.77e-10 t/mm^3^, E = 291 MPa, μ = 0.3, K = 5.7 MPa, N = 0.2741	38
Anterior plate	Hexahedron	Isotropic elastic	E = 110 GPa, μ = 0.3	48
Anterior screw	Hexahedron	Isotropic elastic	E = 110 GPa, μ = 0.3	48

β, viscoelastic parameter; E, Young’s modulus; G, viscoelastic modulus; K, bulk modulus; N, hardening exponent; n, Ci, bi, material constant; μ, Poisson’s ratio.

To validate the post-surgery models, the fused segmental ROM (C4-6) after C5 corpectomy of both post-surgery models were compared with cadaveric experiment results of Hartmann *et al*.^35^ and Zhang *et al*.^26^.

After validation of intact and post-surgery models, we tested both moment-control and displacement-control loading to evaluate the adjacent segment responses. For simulation of all motions, C7 was constrained in six degrees of freedom in the Cartesian coordinate system. A compressive preload of 70 N was given^36^. Bending moments of 0.125 Nm, 0.5 Nm, 1 Nm, 1.5 Nm and 2 Nm were placed on C2 to calculate the adjacent segment (C3-4 and C6-7) ROM and global (C2-7) ROM in flexion, extension, lateral bending and axial rotation. In moment loading, the segmental ROM percentage in the global ROM was compared among post-surgery models with normal and decreased lordosis and the intact model. In displacement loading, a flexion of 30° and extension of 15°^11^ was set to compare the segmental ROM change before and after ACF, as well as the distribution and maximum value of intradiscal pressure at adjacent discs. The LS-DYNA version 971 solver (LSTC, Livermore, CA) was utilized for post-processing.

### Statistical analysis

Statistical analysis was performed using SPSS18.0. To compare the clinical and imaging characteristics between two groups, we compared continuous variables using a paired *t* test and Mann-Whitney U test when appropriate. Categorical variables were compared using Fisher’s exact test, and Pearson’s correlation was used for single factor correlation evaluation. To evaluate the prognostic value of post-surgical lordosis change in predicting RASP occurrence, the Cox regression model was further used after adjustment of age, gender, operation location, fused segment number, pre-operative alignment and operating surgeon. Values of P < 0.05 were considered statistically significant.
